# Stability of BTI Devices for Plasma Rich in Growth Factors (PRGF) Eye Drop Delivery Under Varying Storage and Handling Conditions

**DOI:** 10.3390/biomedicines13051105

**Published:** 2025-05-01

**Authors:** Eduardo Anitua, Iraia Reparaz, María de la Fuente, Mohammad Hamdan Alkhraisat

**Affiliations:** 1 Regenerative Medicine Laboratory, BTI-Biotechnology Institute, 01007 Vitoria, Spain; iraia.reparaz@bti-implant.es (I.R.); maria.delafuente@bti-implant.es (M.d.l.F.); mohammad.hamdan@bti-implant.es (M.H.A.); 2 University Institute for Regenerative Medicine and Oral Implantology—UIRMI (UPV/EHU-Fundación Eduardo Anitua), 01007 Vitoria, Spain; 3 Department of Oral and Maxillofacial Surgery, Oral Medicine and Periodontology Faculty of Dentistry, University of Jordan, Amman 11942, Jordan

**Keywords:** eye drops, blood derivatives, platelet-rich plasma, PRGF, ophthalmic device

## Abstract

**Purpose**: To evaluate the sterility and biological functionality of platelet lysate eye drops stored in BTI ophthalmic devices for PRGF delivery under different storage conditions and simulated use scenarios. **Methods**: Eye drops were prepared using platelet lysate and stored in BTI tubes under three different conditions: ≤−15 °C, 2–8 °C, and room temperature (RT) for 72 h. Simulated use was performed for 72 h with controlled drop dispensing. Bacterial contamination was assessed according to European Pharmacopoeia sterility testing principles. The biological activity of the eye drops was assessed using in vitro proliferation assays with primary human keratocytes (HKs) and human corneal epithelial cells (HCEs). Statistical analyses were performed to compare the effects of different storage conditions and application scenarios. **Results**: No bacterial contamination was detected in platelet lysate eye drops stored under any of the conditions tested, regardless of simulated use. Proliferation assays showed that eye drops enhanced the growth of HK and HCE cells compared to the control medium. No significant differences in proliferation were observed between storage conditions. **Conclusions**: Platelet lysate eye drops maintain sterility and biological functionality when stored in BTI ophthalmic devices at ≤−15 °C, 2–8 °C and RT for up to 72 h of simulated use. These results support the feasibility of using BTI eye drop devices in clinical settings while ensuring safety and efficacy.

## 1. Introduction

Blood derivatives have become a promising therapeutic option for the treatment of eye diseases, offering advantages over conventional artificial eye drops due to their similarity to natural tears [1,2,3,4]. Plasma Rich in Growth Factors (PRGF) is a type of platelet-rich plasma (PRP) derived from autologous blood, which, after activation, coagulation, and retraction, results in an eye drop containing a moderate concentration of plasma and platelet-derived factors and none from leukocytes. PRGF eye drops have demonstrated effectiveness in the treatment of several ocular surface conditions [5,6,7]. The positive clinical outcomes of PRGF eye drops may be attributed to their strong similarity to the natural tear film, as both contain a wide range of proteins and growth factors involved in ocular surface repair [8,9]. Upon activation, platelets release the contents of their granules into the surrounding tissue, initiating a cascade of regenerative and reparative events. Alpha granules and dense granules are two types of specialized organelles found in platelets that act as storehouses for a myriad of bioactive substances. Alpha granules store and release growth factors, cytokines, and chemokines involved in tissue regeneration and wound repair. These granules contain molecules such as platelet-derived growth factor-AB (PDGF-AB) which attracts monocytes, macrophages, and fibroblasts to the injury site, initiating tissue repair; epidermal growth factor (EGF) stimulates the migration and proliferation of corneal cells, enhances DNA synthesis and fibronectin production, promotes mucin-1 secretion by goblet cells, and has anti-apoptotic effects; transforming growth factor-β1 (TGF-β1) favours fibroblast chemotaxis, induces the production of extracellular matrix components like collagen, fibronectin, and proteoglycans and inhibits their degradation, and promotes myofibroblast differentiation, contributing also to anti-inflammatory effects; vascular endothelial growth factor (VEGF) increases vascular permeability and promotes angiogenesis; nerve growth factor (NGF) regulates ocular surface homeostasis in multiple ways; fibronectin supports cell adhesion and migration during corneal repair; and lastly, vitamin A is essential for maintaining epithelial integrity preventing squamous metaplasia among others, which are responsible for a variety of biological pathways involved in ocular tissue regeneration [10,11]. On the other hand, dense granules, also known as beta granules, are rich in procoagulant and adhesive proteins. These granules contain glycoproteins like fibrinogen and von Willebrand factor (vWF), as well as coagulation factors V and XIII. This combination of proteins contributes to platelet aggregation and the stabilization of blood clots. Both types of granules have distinct functions that play an essential role in haemostasis and wound healing. Recently, attention has been focused on the platelets’ released extracellular vesicles (EVs), including exosomes and microvesicles; PRP-EVs promoted cell proliferation and migration and angiogenesis while reducing the inflammatory response, apoptosis, oxidative stress, and senescence. PRP-EVs represent a promising therapeutic approach and a potentially superior cell-free alternative strategy for regenerative medicine [12,13,14].

Additionally, PRGF eye drops share with the tear film essential antimicrobial properties mediated by platelet-derived antimicrobial peptides such as kinocidins and CHDPs (cationic host defence peptides) [15,16], antifibrotic capabilities through the reduction of myofibroblastic markers, and anti-inflammatory effects via immunomodulators, further increasing their therapeutic relevance [17,18,19].

Blood-derived eye drops offer a multifaceted therapeutic approach that goes beyond relieving symptoms. Although corticosteroids and cyclosporin A are cornerstones in the pharmacologic treatment of ocular surface inflammation, their long-term use is often limited by adverse effects. Similarly, nonpharmacologic interventions offer temporary improvements, but no modulation of inflammation or stimulation of tissue repair. PRP-based therapies offer a more comprehensive alternative for sustained ocular surface restoration [3,20]. Unlike advanced preservative-free multi-dose containers, which incorporate one-way valves or sterile filtration membranes to maintain sterility, BTI devices lack a reflux prevention mechanism. This is especially relevant, as conventional multidose systems often rely on preservatives to ensure sterility during prolonged use. However, prolonged exposure to preservatives, especially in chronic treatments, has been associated with ocular surface toxicity including epithelial damage, inflammation, and tear film instability [21,22]. Preservative-free alternatives are preferable for patients with ocular surface disease. BTI vials should be evaluated under simulated clinical conditions due to their open design. However, platelet-derived formulations may not require the same degree of structural protection or chemical preservation as synthetic formulations because of their intrinsic antimicrobial and immunomodulatory properties. This could reduce the risk of contamination even in the absence of complex dispensing systems.

For the effective management of chronic eye diseases, eye drop therapies must retain their biological functionality and stability for several months. To preserve the biological properties of PRGF eye drops in clinical practice, patients are instructed to store them under controlled conditions; vials in use should be maintained at 2–8 °C or at room temperature (RT), while the remaining vials should be stored at ≤−15 °C until required. Previous studies have shown that PRGF eye drops can be stored at ≤−15 °C for up to 12 months without a degradation of key proteins and growth factors involved in ocular surface wound healing. Furthermore, it was shown that PRGF eye drops preserve their composition and biological activity at both 2–8 °C and RT for 7 days after thawing [23].

Another crucial aspect is ensuring the sterility of PRGF eye drops throughout the therapeutic period. In this context, it is essential to assess whether the use of BTI ophthalmic devices with PRGF eye drops during the patient’s treatment period could promote bacterial contamination that might compromise their sterility. Additionally, it is important to assess whether these conditions may influence the stability and therapeutic efficacy of eye drops.

**Figure 1:**
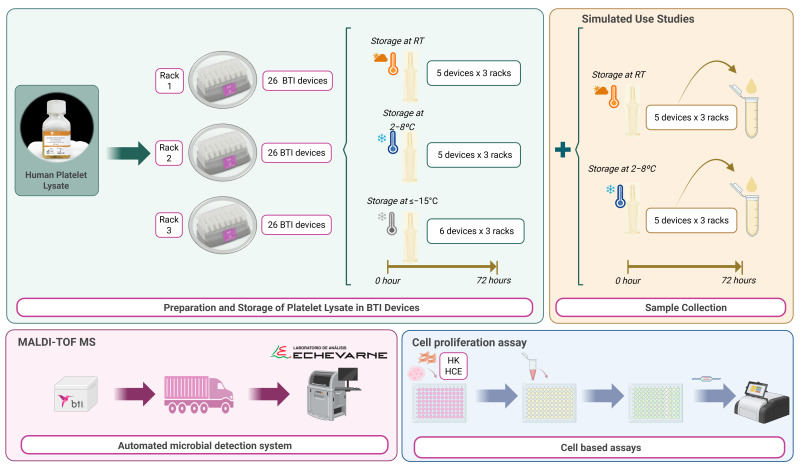
Experimental workflow for preparation, simulated use, and evaluation (bacterial analysis and proliferation assay) of platelet lysate in BTI devices. Created with BioRender.com.

The aim of this study is to determine whether the BTI device is able to maintain both the sterility and the biological potential of PRGF eye drops after simulated use for 48 and 72 h, stored at 2–8 °C and at RT, compared to freshly prepared drops. To achieve this, we chose a commercial human platelet lysate as an experimental model for filling the BTI devices; the proliferative potential and bacterial contamination of the remaining product after the use of BTI devices were assessed under each storage condition and time point (Figure 1).

## 2. Materials and Methods

The biological matrix used for the assays is a fibrinogen-depleted, anticoagulant-free human platelet lysate (ELAREM™ Perform-FD PLUS GMP Grade, PL BioScience, Aachen, Germany). The platelet lysate was thawed at 2–8 °C for 24 h before use. Using a pipette, 9 mL BTI fractionation tubes (TF9) were filled with the platelet lysate inside the hood to avoid external contamination. Once filled, the dispensing of human platelet lysate as a substitute of PRGF-Endoret^®^ (Biotechnology Institute, Vitoria, Spain) eye drops were simulated according to the usual protocol described in the KMU11 ophthalmic kit instructions for use (Biotechnology Institute, Vitoria, Spain), with the manipulation performed outside the laminar flow hood (simulating the clinical protocol with closed technique). For the collection of platelet lysate from TF9 devices, 10 mL plasma tubes (TP10) from ophthalmology kits were used from three different lots: Rack 1 (R1), Rack 2 (R2), and Rack 3 (R3). The racks containing the instruments were also from three different lots. Each device was filled with 0.6 mL of platelet lysate (according to the manufacturer’s instructions). Each rack consisted of 16 devices, distributed according to their purpose and storage conditions. Six units were stored at ≤−15 °C, of which three were for cell culture and three for bacteriological contamination study. Ten devices were stored at room temperature, divided into two for cultures, three for microbiological study and five for simulated use, of which the remaining interior would be used for microbiological study. Finally, a further 10 devices were stored at 2–8 °C, divided into two for cultures, three for microbiological study, and five for simulated use, of which the internal residues would also be used for bacteriological study.

All vials of platelet lysate intended for simulated device use were used for 72 h, the maximum time the devices were used. To analyse the residuals of the devices, 4 drops/day were administered. The devices for bacteriological analysis (MALDI-TOF MS, Matrix-Assisted Laser Desorption Ionization Time of Flight mass spectrometry, Beckman Coulter, Brea, CA, USA) were shipped at 2–8 °C according to the instructions of the destination laboratory (Echevarne Laboratory, Barcelona, Spain).

For each condition, the simulated application was dispensed outside the laminar flow hood into a 1.5 mL microtube. After each dispensing, the cap of the microtube was closed. The microtubes were stored under the same conditions as the stock devices, either at RT or at 2–8 °C and outside the laminar flow hood. Finally (after 72 h), dispensations from the simulated use were collected for cell culture study. For this purpose, the contents of the microtubes were filtered through a 0.22 µm polyethersulfone (PES) filter and stored at ≤−15 °C until further use.

BTI devices with residual platelet lysate eye drops after simulated use were sent to the Echevarne Laboratory for microbiological analysis (MALDI-TOF MS) under Good Laboratory Practice.

Sterility testing was performed according to the European Pharmacopoeia principles for sterility testing. An amount of 0.5 millilitres of each platelet lysate sample stored at different temperatures and used for simulated or non-simulated use was collected for sterility testing. 

To analyse the effect of storage of BTI devices at the different study temperatures, in vitro assays were performed using primary human keratocytes, termed HK (ScienCell Research Laboratories, San Diego, CA, USA), cultured according to the manufacturer’s instructions. Briefly, cells were cultured to confluence in fibroblast medium (FM) supplemented with fibroblast growth supplement (FGS), 2% fetal bovine serum (FBS), and penicillin/streptomycin (complete FM) (ScienCell Research Laboratories, San Diego, CA, USA).

In addition, human corneal epithelial cells transformed with an SV40 adeno vector (HCE-T cells; RRID: CVCL_1272, Riken Cell Bank, Tsukuba, Japan) (referred to as HCE) were cultured in a 5% CO_2_ atmosphere at 37 °C using DMEM/F12 medium (Invitrogen-Gibco, Grand Island, NY, USA) supplemented with 10 ng/mL epidermal growth factor (EGF, Gibco-Invitrogen, Grand Island, NY, USA), 5 mg/mL insulin, 1% DMSO, 7.5% FBS (all from Sigma-Aldrich, St. Louis, MO, USA), and penicillin/streptomycin. At confluence, cells were detached using an animal-free trypsin-like enzyme (TrypLE Select, Gibco-Invitrogen, Grand Island, NY, USA). Cell viability was assessed by the trypan blue dye exclusion method.

HK cells and HCE cells were seeded at a density of 6000 cells/cm^2^ and 20,000 cells/cm^2^, respectively, in black 96-well optical bottom microplates using serum-free medium supplemented with 10% and 5% (vol/vol), respectively, of the sample pool collected after simulated use of platelet lysate obtained from BTI devices and maintained at each of the above time/temperature conditions for 72 h. Cell density was analysed using the CyQUANT cell proliferation assay (Invitrogen, Carlsbad, CA, USA). Briefly, the culture medium was removed, and the wells were carefully washed with phosphate-buffered saline (PBS) to avoid disturbing the cell monolayer. The plate was then frozen at −80 °C to improve the efficiency of cell lysis. It was then thawed at room temperature and the CyQUANT assay (Thermo Fisher Scientific, Waltham, MA, USA) was performed according to the manufacturer’s instructions, including RNase A treatment. The fluorescence of the samples was measured using a multimode reader (BioTek Synergy H1 Agilent Technologies, Santa Clara, CA, USA) and the results were expressed in fluorescence units (FUs). An internal control for the basal cell growth of each phenotype used in the study was included in each plate consisting of the routine medium of each phenotype. To normalise the cell growth of samples incubated in different plates, the fluorescence value obtained in each well with the platelet lysate sample was divided by the mean value of the corresponding control in the same plate (ratio = platelet lysate sample/control).

The data obtained were expressed as mean ± standard deviation. ANOVA was used to analyse the possible differences between the control and the stored platelet lysates (≤−15 °C, room temperature, and 2–8 °C and the conditions of use or non-use). The general linear repeated measures model was used to compare platelet lysates stored at different temperatures and use conditions. The Bonferroni correction was used as post-hoc analysis. Statistical analyses were performed using SPSS software (version 15.0; SPSS Inc., Chicago, IL, USA) with a significant level of 95%.

## 3. Results

**Figure 2:**
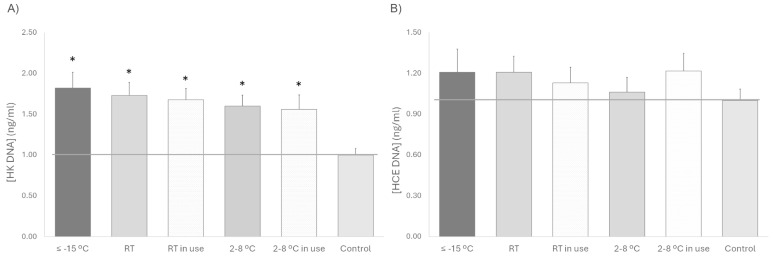
Cell proliferation for 72 h (**A**) HK and (**B**) HCE treated with platelet lysate stored at different temperatures (≤−15 °C, RT and 2–8 °C) and with or without sham application (in use). The gray line, which is set at one unit, helps to visualize the control reference. * *p* < 0.05 respecting the control.

In terms of sterility, no bacteriological contamination was detected in any of the devices tested at different temperatures, simulated use or non-use. In addition, no evidence of microbial growth was observed in any of the cell cultures used to study proliferation with the different platelet lysate under any storage condition. In the proliferation assay, the proliferative response of HK cells was significantly increased after treatment with platelet lysate compared to the control (standard medium), being very similar under all conditions, but it tended to be slightly lower when stored at 2–8 °C in comparison to samples that remained frozen at ≤−15 °C. In the case of HCE, the cellular response to platelet lysates stored under the different conditions and either used or not in use was very similar, with no differences compared to the control. No significant differences in the proliferative response to platelet lysates stored at the three temperatures tested (≤−15 °C, RT, or 2–8 °C) were observed for any of both cell phenotypes. However, for HK cells, a trend towards lower proliferation was observed for used platelet lysates compared to unused platelet lysates stored at the same temperature (Figure 2).

## 4. Discussion

This study assessed the biological activity and microbial contamination of BTI devices stored at room temperature or 2–8 °C for 72 h under simulated conditions of use. Previous studies have demonstrated the efficacy of PRGF eye drops in treating various ocular surface disorders including dry eye [24], Sjögren’s syndrome [25], and neurotrophic keratitis [26]. These eye drops are produced following a standardized protocol, ensuring greater consistency compared to other blood-derived products [27].

The manufacture of PRGF eye drops is in accordance with the regulatory framework published on the 23 May 2013 by the Spanish Agency for Medicines and Health Products, which establishes the classification of the non-replacement therapeutic use of autologous plasma and its fractions, components, or derivatives as medicinal products for human use to meet special needs. Given their therapeutic potential and standardized production, ensuring proper storage conditions becomes crucial. Notably, PRGF eye drops can be stored at ≤−15 °C for up to 12 months without compromising key growth factors and proteins necessary for ocular surface tissue regeneration, and without the risk of microbial contamination. Furthermore, their biological activity remains intact when stored at 2–8 °C or RT for 3 to 7 days. The present study, importantly, extends these findings by demonstrating that platelet lysate remains both biologically active and free of bacterial contamination after 72 h of use under simulated clinical conditions, both at room temperature and refrigerated.

The current results have substantial clinical relevance in the context of ocular surface disease management. The demonstrated preservation of biological activity and sterility of the platelet derivative under both refrigerated and ambient conditions for up to 72 h directly supports their practical application in real-world clinical settings. This evidence provides a basis for reconsidering current storage and handling recommendations within clinical guidelines, potentially allowing greater flexibility without compromising therapeutic efficacy or patient safety. Although many ophthalmic eye drops are packaged in sterile containers, they often include preservatives to maintain the sterility after opening. However, contamination can still occur due to improper handling by patients, carers, and healthcare professionals; for example, direct contact with fingers or eyelids is a risk for contamination of the dropper tip, which can introduce pathogens into the bottle, increasing the chance of eye infection and inflammation [28]. Additionally, ambient air entering the container can also contribute to microbial contamination. One approach to maintain the sterility of the composition is to use an added preservative [21]. Historically, the most used preservative has been benzalkonium chloride (BAK). BAK is a detergent that acts by disrupting the lipid layer of the cell membrane of the tear film, leading to its instability and exerting cytotoxic effects on the ocular surface and various other structures in the eye apart from triggering inflammatory responses [29]. A multicentre study involving 9658 glaucoma patients who used both preservative and preservative-free eye drops showed that those who used the latter had significantly fewer ocular symptoms and signs of irritation compared to those who used preservative eye drops [30]. A recent study showed that the use of BAK-preserved artificial tears was associated with an increased probability of experiencing the symptoms of dry eye disease, blurred vision and itching, and a tendency to declare a higher number of symptoms when using a BAK-preserved artificial tear, compared with the use of non-preserved artificial tears [31]. Preservative-free products are the preferred choice for both patients and healthcare professionals, as there is a clinical tendency to avoid their use due to a fundamental concern about the safety and effects of preservatives in ophthalmic therapies [22,32,33]. The desired solution is an affordable and easy-to-use dispensing system that ensures product sterility; in this regard, it is crucial to assess whether the dispensing device or the formulation itself contains features that prevent contamination, as these products are particularly susceptible to contamination from repeated handling by patients. In this sense, we evaluated the sterility and biological activity of a commercial platelet lysate dispensed in BTI vials after simulated use and storage under different temperature conditions. Our results show that no bacterial contamination was detected in any of the eye droppers containing platelet lysate eye drops tested at each time point and under all temperature conditions evaluated during use. Furthermore, corneal epithelial and keratocytes cultures treated with a pool of material collected during the use of platelet lysate eye drops showed no microbial contamination. Moreover, recent studies have highlighted the natural antimicrobial properties of blood-derived products [34,35].

Therefore, the stability of PRGF eye drops combined with the sterile BTI dispensing system provides a preservative-free while safe option for ocular surface therapy, even under conditions of prolonged and repeated patient use.

Another important clinical implication is the resistance of PRGF formulations to temperature changes during transportation or patient handling. Our study shows that neither refrigeration (2–8 °C) nor storage at RT for up to 72 h significantly altered the biological activity or sterility of the product. Importantly, such robustness reduces the dependence on a strict cold chain, which is particularly beneficial for patients living in remote or low-resource settings and may influence future recommendations for PRGF handling in clinical guidelines. In addition, the biological potential of platelet lysate is not affected by the storage at different temperatures, making it easier for patients to use. In all cases, it is important to follow proper handling procedures, such as washing hands before each application and avoiding contact with the tip, to ensure product safety and adequate storage. BTI devices are good eye drop dispensers with no risk of bacterial contamination during use, whether stored in a refrigerator or at room temperature, while maintaining biological activity.

## 5. Conclusions

In conclusion, this study demonstrates that BTI devices remain free of microbial contamination when stored at room temperature or between 2 and 8 °C and subjected to simulated use, while maintaining the biological activity of the platelet lysate. These results confirm the sterility, stability, and functional integrity of the system under conditions that closely mimic clinical handling. Although clinical outcomes were not evaluated, these results provide important translational evidence to support the safe and effective use of BTI devices in ophthalmic applications where maintaining the regenerative potential of blood-derived formulations is essential.

## Data Availability

The raw data supporting the conclusions of this article will be made available by the authors on request.

## Abbreviations

The following abbreviations are used in this manuscript:

PRGF	Plasma Rich in Growth Factor
BTI	Biotechnology Institute
RT	Room Temperature
HK	Human Keratocytes
HCE	Human Corneal Epithelial cells
PRP	Platelet Rich Plasma
PDGF-AB	Platelet Derived Growth Factor-AB
EGF	Epidermal Growth Factor
TGF-β1	Transforming Growth Factor beta-1
VEGF	Vascular Endothelial Growth Factor
NGF	Nerve Growth Factor
vWF	Von Willebrand Factor
EV	Extracellular Vesicles
CHDP	Cationic Host Defense Peptides
TF9	9 mL BTI Fractionation tube
TP10	10 mL Plasma tube
PES	Polyethersulfone
FM	Fibroblast Medium
FGS	Fibroblast Growth Supplement
FBS	Fetal Bovine Serum
PBS	Phosphate-buffered saline
FU	Fluorescence Unit
BAK	Benzalkonium Chloride
